# Beyond cannabinoids: Application of NMR-based metabolomics for the assessment of *Cannabis sativa* L. crop health

**DOI:** 10.3389/fpls.2023.1025932

**Published:** 2023-03-22

**Authors:** Santiago Fernández, Rossina Castro, Andrés López-Radcenco, Paula Rodriguez, Inés Carrera, Carlos García-Carnelli, Guillermo Moyna

**Affiliations:** ^1^ Laboratorio de Farmacognosia y Productos Naturales, Departamento de Química Orgánica, Facultad de Química, Universidad de la República, Montevideo, Uruguay; ^2^ Laboratorio de Fisicoquímica Orgánica, Departamento de Química del Litoral, Centro Universitario Regional Litoral Norte, Universidad de la República, Paysandú, Uruguay; ^3^ Laboratorio de Biocatálisis y Biotransformaciones, Departamento de Química Orgánica and Departamento de Biociencias, Facultad de Química, Universidad de la República, Montevideo, Uruguay; ^4^ Laboratorio de Experimentación Animal – Área Farmacología, Departamento de Ciencias Farmacéuticas, Facultad de Química, Universidad de la República, Montevideo, Uruguay

**Keywords:** cannabinoids, Cannabis sativa, NMR-based metabolomics, powdery mildew, chemovar

## Abstract

While *Cannabis sativa* L. varieties have been traditionally characterized by their major cannabinoid profile, it is now well established that other plant metabolites can also have physiological effects, including minor cannabinoids, terpenes, and flavonoids. Given the multiple applications of cannabis in the medical field, it is therefore critical to characterize it according to its chemical composition (i.e., its metabolome) and not only its botanical traits. With this in mind, the cannabinoid and metabolomic profiles from inflorescences of two *C. sativa* varieties with either high Δ^9^-tetrahydrocannabinolic acid (THCA) or high cannabidiolic acid (CBDA) contents harvested at different times were studied. According to results from HPLC and NMR-based untargeted metabolomic analyses of organic and aqueous plant material extracts, we show that in addition to expected variations according to cannabinoid profiles, it is possible to distinguish between harvests of the same variety. In particular, it was possible to correlate variations in the metabolome with presence of powdery mildew, leading to the identification of molecular markers associated with this fungal infection in *C. sativa*.

## 1 Introduction

Cannabis (*Cannabis sativa* L.) is a widely distributed plant that has been a source of fiber, food, oil, and medicines for millennia (Clarke and Merlin, 2016). The first reports regarding its medicinal use date back to the third millennium BC in ancient China (Wills, 1998). Since then, several cultures have employed cannabis preparations to treat a variety of ailments (Russo, 2014). Since our understanding of its therapeutic potential is continuously improving, the interest in cannabis as a source of compounds for use in medicinal preparations has increased notably (Procaccia et al., 2022). Furthermore, the non-pharmacological uses of the plant and its derivatives, particularly as a source of fibers and as a recreational drug, are well known.

*C. sativa* produces an important number of secondary metabolites, and more than 500 compounds have been identified in cannabis so far (ElSohly and Gul, 2014). In particular, cannabinoids, terpenoids, and flavonoids stand out (ElSohly and Slade, 2005). Of the three compound classes, the former are of great relevance given their biological activity and the fact that they are almost exclusively found in this plant (Hanuš et al., 2016; Gülck and Møller, 2020). Most of the known pharmacological properties are associated to these naturally-occurring compounds, particularly Δ^9^-tetrahydrocannabinol (THC) and cannabidiol (CBD). These compounds are present mostly as carboxylic acids in the plant, namely Δ^9^-tetrahydrocannabinolic and cannabidiolic acids (THCA and CBDA), and are transformed to the neutral molecules through non-enzymatic decarboxylation (Flores-Sanchez and Verpoorte, 2008). However, the biological effects of *C. sativa* do not depend only on the levels of THC and CBD (Procaccia et al., 2022). Indeed, nearly 150 phytocannabinoids which can be classified in different structural subclasses have been isolated from this plant (Hanuš et al., 2016), and several of these compounds, sometimes referred to as minor cannabinoids, have pharmacological potential (Franco et al., 2020). Furthermore, a number of studies suggest that the interaction or synergy between these cannabinoids and other secondary metabolites leads to more pronounced effects than those produced by the isolated compounds (Russo, 2011; Blasco-Benito et al., 2018; Russo, 2019; Maayah et al., 2020; Goerl et al., 2021). In addition, the botanical diversity of *C. sativa* has resulted in longstanding discussions regarding its taxonomical characterization. It is now generally accepted that the plant is a single species that can be classified in different subspecies and varieties (Small, 2015; McPartland, 2018). Independently of the names and botanical traits used to refer to it, the chemical composition is what will determine the pharmacological usefulness of the plant. Therefore, several authors stress that the categorization of *C. sativa* varieties should be done according to its chemical profile (Russo, 2019), and refer to them as chemical varieties or chemovars (Lewis et al., 2018).

In addition to secondary metabolite content, the quality of a medicinal plant is also given by the absence of contaminants and adulterants. Natural contaminants are usually introduced during cultivation and storage, and consist of degradation products, microbial contamination, and heavy metals (American Herbal Pharmacopoeia, 2014). In particular, fungi can colonize different organs of the plant, causing a reduction in the quality of the products (Punja et al., 2019). Furthermore, the consumption of a contaminated product can cause harmful effects on the health of consumers (Montoya et al., 2020). Microbial infections can also lead to biotic stress, which can alter the secondary metabolite composition (Gorelick and Bernstein, 2017).

While the determination of the main cannabinoid content is a required assay for cannabis products that rely on the biological activity of the plant extract, comprehensive knowledge of the profile of other secondary metabolites in these materials is also relevant (Jin et al., 2021). Thus, establishing the metabolic fingerprints can provide important information to assure the composition and quality of a given *C. sativa* chemovar (Puri et al., 2021). Variations in this profile can indicate that the material is not appropriate for its intended uses. In particular, the discrimination between chemovars can be conveniently carried out through NMR-based metabolomic analysis (Choi et al., 2004a). Indeed, a recent review concludes that metabolomics represents an ideal bioanalytical tool that could greatly assist and accelerate cannabis research and development, even coining the term “cannabinomics”(Aliferis and Bernard-Perron, 2020).

In the present report we carry out an exhaustive characterization of a high-THCA and a high-CBDA *C. sativa* chemovars, both with medicinal potential, through the determination of metabolite profiles complemented with basic information derived from cannabinoid composition. Briefly, our results not only allowed us to clearly distinguish between the two plant varieties, but also to identify a crop with a microbial infection that, interestingly, does not alter the main cannabinoid profile and yield in a relevant way. Furthermore, through metabolomic analysis it was possible to identify variations in specific metabolites that could shed light on infection mechanisms.

## 2 Materials and methods

### 2.1 Chemicals and reagents

Analytical grade chloroform and methanol were from Dorwil (Buenos Aires, Argentina), HPLC grade acetonitrile was obtained from Carlo Erba (Val-de-Reuil, France), and formic acid, choline (> 98%), deuterochloroform (CDCl_3_, 99.8%), and deuterium oxide (D_2_O, 99.9%) were purchased from Merck (Kenilworth, NJ, USA). Betaine (> 98%) was from Thermo Fisher Scientific (Geel, Belgium). Diazepam (> 99.9%) and standard solutions of CBDA, CBD, cannabichromene (CBC), cannabinol (CBN), cannabigerol (CBG), THCA, THC, and Δ^8^-THC at 1 mg/mL were purchased from Lipomed (Cambridge, MA, USA). Standard solutions of Δ^9^-tetrahydrocannabivarinic acid (THCVA), Δ^9^-tetrahydrocannabivarin (THCV), cannabigerolic acid (CBGA), cannabidivarinic acid (CBDVA), cannabidivarin (CBDV) and cannabichromenic acid (CBCA) at 1 mg/mL were purchased from Cerilliant (Round Rock, TX, USA).

### 2.2 Plant material and sample preparation

*Cannabis sativa* L. female inflorescences were donated by Khiron Life Sciences Uruguay S.A. and employed in compliance with guidelines from a scientific research license granted by the Instituto de Regulación y Control del Cannabis (IRCCA). Plant material from a drug chemotype high in THCA (chemovar A) and a fiber chemotype high in CBDA (chemovar B) were used. Plants were grown under identical indoor conditions, which consisted of temperature and relative humidity ranges of 24-28 °C and 60-70% for the vegetative stage, and 20-26 °C and 55-70% for the flowering stage. Samples were obtained from three different harvests for each chemovar. In the case of chemovar A, these were carried out on March, June, and September 2020, while for chemovar B harvests were on March, June, and December 2020.

Samples for cannabinoid composition determinations were dried at 35 °C for 24 h and ground with a manual herb grinder. Once a particle size range from 0.5 to 2 mm was obtained, 500 mg were extracted with a 9:1 methanol-chloroform mixture by dynamic maceration for 30 min at room temperature, followed by sonication for 15 min. The extract was then filtered and diluted with methanol to a final volume of 50 mL. A 100 μL aliquot of this solution was evaporated under a nitrogen stream and redissolved in 100 μL to 1 mL of acetonitrile containing diazepam (10 μg/mL) as an internal standard. The resulting solutions were employed in chromatographic analyses using the conditions detailed below. These extractions were performed in duplicate.

For NMR-based metabolomic analyses, 20 samples per chemotype from each harvest were collected, frozen in liquid nitrogen, ground manually using a glass rod, and stored at -20 °C. Extractions were carried out following a method originally proposed by Choi and coworkers (Choi et al., 2004a). Briefly, 500 mg of each sample were suspended in 10 mL of a 1:1:2 methanol-water-chloroform mixture, subjected to vortex agitation for 30 s, sonicated for 1 min, and then centrifuged at 3000 rpm for 20 min. Phases were separated and the extraction of the plant material was repeated. Following phase separation, organic solvents were removed in a rotary evaporator under vacuum at 35 °C, while aqueous phases were dried by lyophilization. Two dry extracts per sample were obtained.

### 2.3 HPLC analysis

The main cannabinoid composition was determined using a recently reported method (Fernández et al., 2022), using a Shimadzu DGU-205R Ultra HPLC-PDA system equipped with a Hypersil C18 (150 × 4.6 mm, 3 μm particle size) column and a Hypersil BDS C18 (10 × 4 mm, 3 μm particle size) precolumn (ThermoFisher Scientific, Waltham, MA, USA). Linear calibration curves (r^2^ > 0.99) for all cannabinoids analyzed were constructed in the range of 1 to 100 μg/mL. The column oven was set to 25 °C, the injection volume was 10 μL, and the total run time was 18 min using a solvent gradient composed of 1.0 mM formic acid buffer at pH 3.53 (solvent A) and acetonitrile (solvent B) as mobile phase at 1 mL/min flow. The gradient started with 70% B for 7 min, then increased to 83.5% B and held constant for 1 min, then increased to 88.5% B over a 3 min period, 1 additional min to reach 99% B, then kept constant for 2 min followed by a 2 min to lower B to 70%, and finally held constant for 2 min. This method allowed the separation of the 14 cannabinoids available from a mix of standards.

### 2.4 NMR experiments

Organic extracts were dissolved in 0.7 mL of CDCl_3_ and aqueous extracts in 0.7 mL of D_2_O, and then transferred to 5 mm NMR tubes (New Era Enterprises Inc., Vineland, NJ, USA). All NMR spectra were recorded at 25 °C on a Bruker AVANCE III 500 NMR spectrometer operating at ^1^H and ^13^C frequencies of 500.13 and 125.76 MHz, respectively, and equipped with a *z*-gradient TXI probe. A spectral width of 10 KHz, a data size of 32 K, and a total of 64 scans were employed to record 1D spectra, using a relaxation delay of 4 s between scans. Regular 1D acquisition sequences with 30^o^ excitation pulses were employed, using a standard water presaturation scheme with aqueous samples. When required, 1D-TOCSY, 1D-NOESY, HSQC, and HMBC spectra were acquired and processed using acquisition and processing parameters provided with the spectrometer.

To confirm the presence of choline and betaine in the aqueous extracts, 10 μL aliquots from 50 mM standard solutions of each compound were added to selected samples, and the 1D ^1^H NMR experiments repeated under the same conditions described above.

### 2.5 NMR data processing

NMR data for metabolomic analyses were processed and analyzed with MNova (version 12.0, MestreLab Research, S.L., Santiago de Compostela, Spain). Free induction decays were zero-filled to 64 K points and apodized with a 0.3 Hz exponential window function prior to Fourier transformation. All spectra were manually phase- and baseline-corrected and referenced. In the case of organic extracts, the residual CHCl_3_ solvent signal at 7.28 ppm was used for calibration, while the anomeric proton signal of α-glucose at 5.22 ppm was employed in aqueous samples. Spectra corresponding to the same class of extract were aligned using PAFFT (Wong et al., 2005), and the data was normalized to the total spectral area after excluding residual solvent resonances and regions without signals. The resulting data matrices were finally exported as text files for use in statistical analyses.

### 2.6 Multivariate statistical analysis

Multivariate statistical analyses, including principal component analysis (PCA) and orthogonal partial least squares discriminant analysis (OPLS-DA), were carried out with the PLS_Toolbox package (version 8.5, Eigenvector Research Inc., Manson, WA, USA) implemented for MATLAB (revision 2014a, The MathWorks Inc., Natick, MA, USA). For all models, the data was mean-centered and scaled using a Pareto factor (van den Berg et al., 2006). Analysis of the data was first performed with PCA, which reduces the dimensionality and facilitates the identification of data clusters or trends (Wold et al., 1987; Trygg and Wold, 2002; Trygg et al., 2007). The PCA scores plot was also employed to identify strong outliers outside the 95% significance region of Hotelling’s T2 ellipse. Cross-validation of OPLS-DA models was achieved using the random subset method, which involved 20 iterations over data split into 10 equally-sized parts. Receiver operating characteristic (ROC) curves were plotted, and areas under the curves were calculated to ensure the goodness of fit of the resulting models (Ekelund, 2012; Simundic, 2012). Permutation tests with 100 iterations were also performed to determine the degree of over-fitting and further validate the discriminant analyses (Ni et al., 2008). When needed, statistical total correlation spectroscopy (STOCSY) analyses were performed with an in-house MATLAB script based on the algorithm described by Cloarec and coworkers (Cloarec et al., 2005).

### 2.7 Fungi characterization

The fungal infection was evidenced by the presence of off-white powdery spots typical of powdery mildew disease. Microscopic examination of fungi was carried out with a Nikon Eclipse E100 optical microscope. To examine asexual morphs, a piece of clear adhesive tape was placed on infected leaves, stripped off, and placed on a microscope slide with one drop of distilled water. The observations were done at magnifications of 40, 100, and 400 under standard light. Germinated conidia were examined on fungi grown with potato dextrose agar medium.

## 3 Results

**Tables 1**, **2** show the cannabinoid composition of chemovars A and B. As expected, the two are notably different, and characterized, respectively, by their particularly high content of THCA and CBDA. Moreover, the acidic forms of other cannabinoids are also predominant, and CBN was only detected in one sample and below the quantification limits. This suggests that little degradation occurred during sample handling and analysis. For chemovar A, the total cannabinoid content ranged from 15.14 ± 0.50 to 16.70 ± 1.10%, with a mean of 15.50 ± 1.10% (n = 6, two samples per crop, three crops). Similarly, the cannabinoid content in chemovar B crops ranged from 16.50 ± 0.43 to 18.36 ± 0.08% with a mean of 17.60 ± 0.80% (n = 6, two samples per crop, three crops). These results agree with literature reports, which indicate average cannabinoid contents in inflorescences ranging from 5 to 25% (Jin et al., 2021). Inspection of the results also shows that relative cannabinoid composition and total cannabinoid yield is stable through the crops in both chemovars.

**Table 1:** Composition of chemovar A plant material (mean ± SD)^a^.

Compound	Crop 1	Crop 2	Crop 3
CBDVA	nd^b^	nd	Nd
CBDV	nd	nd	nd
CBDA	0.27 ± 0.01	0.36 ± 0.03	0.04 ± 0.01
CBGA	0.081 ± 0.005	0.080 ± 0.003	0.11 ± 0.01
CBG	0.091 ± 0.002	0.066 ± 0.001	0.12 ± 0.01
CBD	nd	nd	nd
THCV	nd	nd	< 0,02
THCVA	0.068 ± 0.001	0.057 ± 0.004	0.14 ± 0.01
CBN	nd	nd	< 0.02
Δ^9^-THC	0.659 ± 0.007	0.272 ± 0.002	0.390 ± 0.042
Δ^8^-THC	nd	nd	< 0.02
CBC	0.01 ± 0.01	< 0.02	nd
THCA	13.40 ± 0.50	14.97 ± 0.12	15.1 ± 1.1
CBCA	0.56 ± 0.02	0.273 ± 0.003	0.770 ± 0.001
Total^c^	15.14 ± 0.50	16.07 ± 0.12	16.70 ± 1.09

a Data are expressed as weight of cannabinoid per 100 g of dried inflorescences (wt%).

b not detected.

c Total cannabinoids expressed as the sum of cannabinoid weights per 100 g of dried inflorescences (wt%).

**Table 2:** Composition of chemovar B plant material (mean ± SD)^a^.

Compound	Crop 1	Crop 2	Crop 3
CBDVA	< 0.02	< 0.02	< 0.02
CBDV	nd^b^	nd	nd
CBDA	14.30 ± 0.04	14.38 ± 0.10	13.23 ± 0.40
CBGA	0.080 ± 0.007	0.10 ± 0.01	0.100 ± 0.002
CBG	< 0.02	< 0.02	< 0.02
CBD	0.86 ± 0.01	0.23 ± 0.02	0.48 ± 0.01
THCV	nd	nd	nd
THCVA	nd	nd	nd
CBN	nd	nd	nd
Δ^9^-THC	0.070 ± 0.007	0.030 ± 0.002	0.057 ± 0,004
Δ^8^-THC	nd	nd	< 0.02
CBC	0.23 ± 0.01	0.066 ± 0.005	0.14 ± 0.01
THCA	0.87 ± 0.06	0.58 ± 0.04	0.26 ± 0.01
CBCA	1.97 ± 0.02	2.41 ± 0.08	2.2 ± 0.2
Total^c^	18.36 ± 0.08	17.79 ± 0.14	16.50 ± 0.43

a Data are expressed as weight of cannabinoid per 100 g of dried inflorescences (wt%).

b not detected.

c Total cannabinoids expressed as the sum of cannabinoid weights per 100 g of dried inflorescences (wt%).

**Figure 1:**
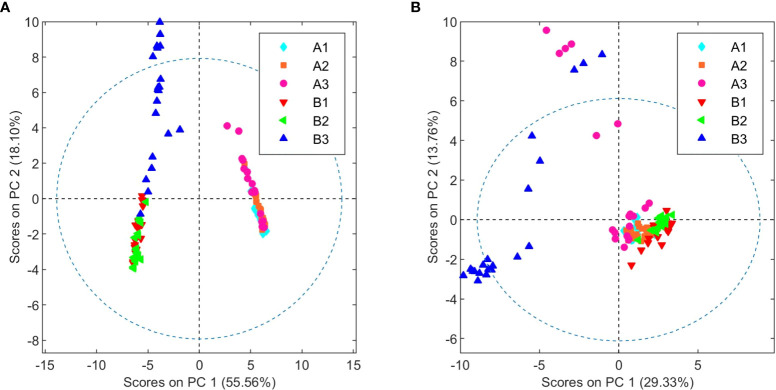
PCA score plots of organic **(A)** and aqueous **(B)** extracts from chemovars A and B colored according to harvest.

Following the determination of the basic cannabinoid composition, the metabolic profiles for both *C. sativa* varieties were investigated. For this purpose, 20 samples of each chemovar from the three harvests considered were collected, resulting in a total of 60 organic and 60 aqueous extract samples. PCA score plots obtained from the ^1^H NMR data recorded for the two sets are shown in **Figure 1**. It is clear that both chemovars can be easily differentiated on the basis of NMR data from their organic or aqueous extracts. Indeed, the first two PCs account for 73.7% of the variance in the organic set, with samples from chemovar A forming a cluster showing positive PC1 scores, whereas chemovar B samples have negative scores on this PC. Similarly, only two PCs were sufficient to account for nearly 90% of the variance in the aqueous data set.

**Figure 2:**
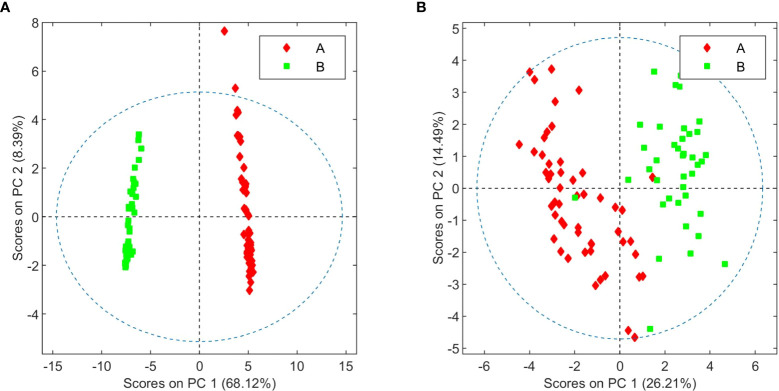
PCA score plots of organic **(A)** and aqueous **(B)** extracts from chemovars A and B after removal of outliers.

Cursory inspection of both PCA plots indicates that six of the aqueous samples corresponding to the third crop of chemovar A deviate from the others, suggesting errors during the preparation of these samples. On the other hand, all 20 organic and aqueous extracts from the third crop of chemovar B deviate considerably from the rest. As discussed below, this is likely associated with fungal infections affecting plants from this particular crop. Therefore, the PCA score plots were recomputed without the outliers from both chemovars. As shown in **Figure 2**, ^1^H NMR profiles from organic and aqueous extracts can still differentiate the two chemovars clearly.

**Figure 3:**
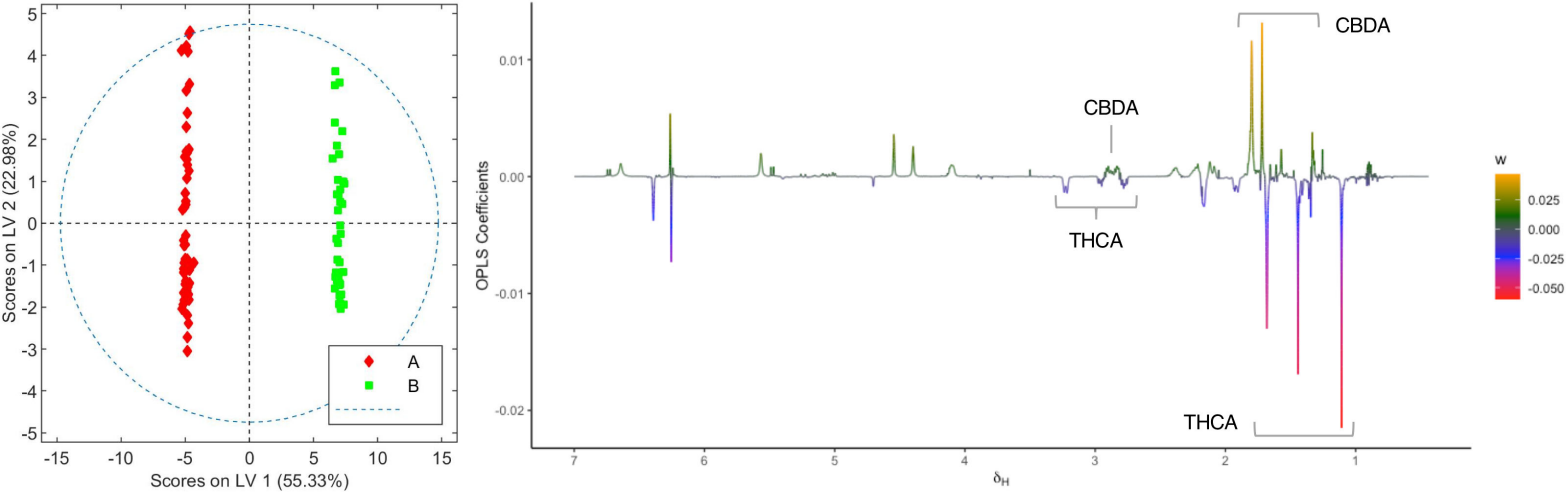
Score and loading factor plots obtained from the OPLS-DA between organic extracts of chemovars A and B. The metabolites that differentiate the two groups are annotated in the loading factor plots. The R^2^Y and Q^2^Y coefficients for the model were 0.99 and 0.99, respectively, and the ROC curve had an AUC value of 1.00 (see **Figures S1**, **S2**).

In order to identify the metabolites that differentiate the two chemovars, OPLS-DA models from the depurated organic and aqueous extract ^1^H NMR datasets were generated. Inspection of the OPLS-DA loading factor plot obtained from organic extracts indicates that signals at 1.44, 1.25, and 1.11 ppm, corresponding to THCA, correlate to chemovar A, while signals for CBDA at 1.80 and 1.72 ppm corroborate that the levels of this cannabinoid are higher in chemovar B (**Figure 3**) (Choi et al., 2004b). These results are not surprising and are consistent with those obtained by the HPLC analyses presented in **Tables 1**, **2**.

**Figure 4:**
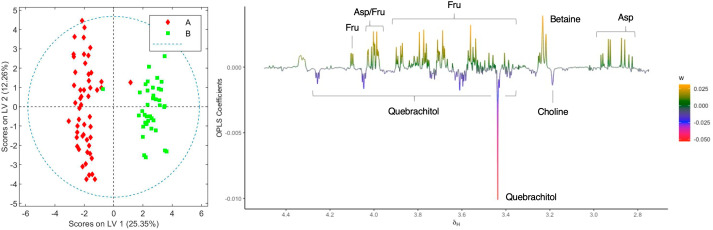
Score and loading factor plots obtained from the OPLS-DA between aqueous extracts of chemovars A and B. The metabolites that differentiate the two groups are annotated in the loading factor plots. The R^2^Y and Q^2^Y coefficients for the model were 0.91 and 0.83, respectively, and the ROC curve had an AUC value of 0.98 (see **Figures S9**, **S10**).

When the loading factor plot of the OLPS-DA between aqueous extracts of the two chemovars was analyzed (**Figure 4**), positive signals at 4.10, 4.03-3.97, 3.88, 3.82-3.77, 3.70-3.63, and 3.59-3.52 ppm, and at 3.99, 2.93, and 2.84 ppm, which can be respectively assigned to fructose and aspartate by comparison to literature data (Wishart et al., 2009; Barclay et al., 2012; de Falco et al., 2016), show that the levels of these two metabolites are higher in chemovar B plants. In addition, a positive signal at 3.23 ppm with an HSQC correlation to a ^13^C resonance at 53.4 ppm likely corresponds to the *N-*methyl groups of betaine (de Falco et al., 2016; Wishart et al., 2022), indicating that the levels of this trimethylammonium-bearing metabolite are also higher in the high-CBDA chemovar specimens (**Figure S3**). This initial assignment was confirmed by spiking one of the aqueous extract samples with a betaine standard solution (see **Figure S4**). On the other hand, negative peaks at 4.24, 4.04, 3.73, 3.59, 3.57, 3.44, and 3.38 ppm match nicely with reference data for the carbasugar quebrachitol (De Almeida et al., 2012). This reveals that the concentration of this cyclitol, whose identity was further established by means of STOCSY, 1D-TOCSY, 1D-NOESY, and heteronuclear correlation NMR experiments (see **Figures S5**–**S7**; **Table S1**), is higher in chemovar A plants. Moreover, a negative singlet at 3.18 ppm correlating to a ^13^C resonance at 53.9 ppm in the HSQC spectrum corresponds to choline (de Falco et al., 2016; Wishart et al., 2022), suggesting that the levels of this quaternary aminoalcohol are higher in the high-THCA *C. sativa* variety (**Figure S8**). As was the case for betaine, this assignment was confirmed unequivocally by spiking experiments (**Figure S4**).

**Figure 5:**
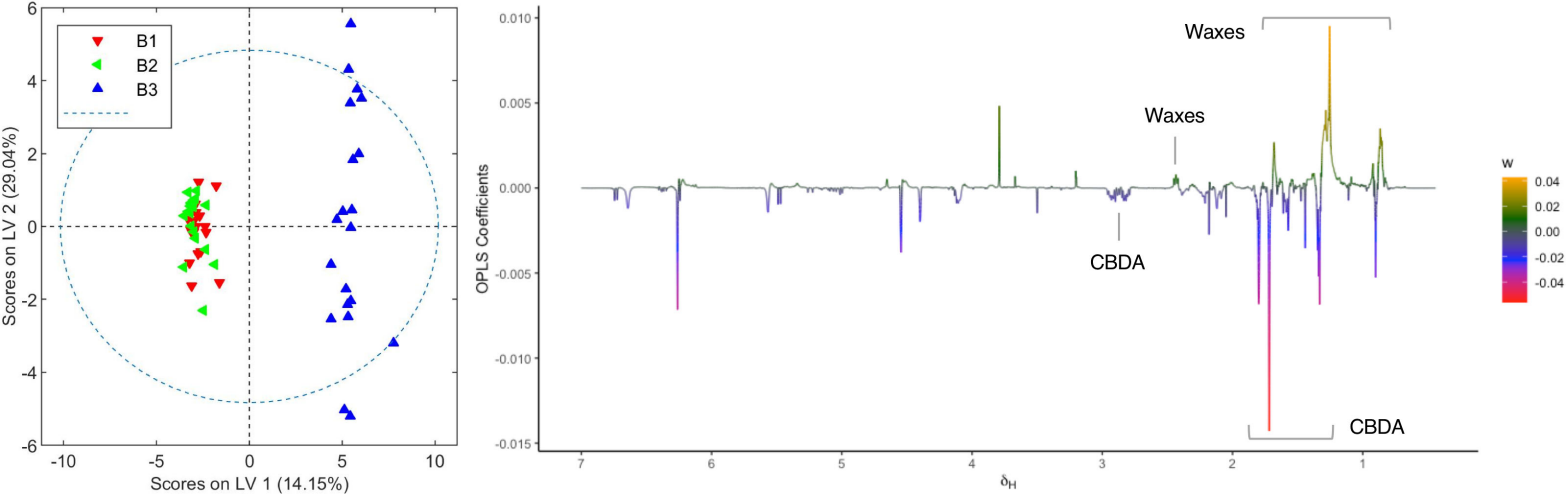
Score and loading factor plots obtained from the OPLS-DA between organic extracts of healthy and infected crops of chemovar B. The metabolites that differentiate the two groups are annotated in the loading factor plots. The R^2^Y and Q^2^Y coefficients for the model were 0.98 and 0.91, respectively, and the ROC curve had an AUC value of 1.00 (see **Figures S13**, **S14**).

Plants from the third crop of chemovar B showed signs of powdery mildew infection. The morphological characteristics of this fungal leaf growth and its asexual microscopic structures allowed us to classify it as a member of the *Golovinomyces* genus (**Figures S11**, **S12**). Since this infection was also clearly evidenced in the initial clustering analyses depicted in **Figure 1**, we decided to carry out a more exhaustive comparison between healthy and infected plants within this chemovar using OPLS-DA. As shown in **Figure 5**, the model obtained with ^1^H NMR data from organic extracts easily differentiates inflorescences of the first two crops (B1+B2) from those of the third (B3). The corresponding loading factor plot identifies CBDA as one of the discriminating metabolites in this case, and, in agreement with HPLC findings presented in **Table 2**, indicates that higher concentrations of this cannabinoid are present in healthy plants of the high-CBDA chemovar. On the other hand, higher levels of waxes, with signals at 2.42, 1.70, 1.28-1.25, and 0.92 ppm (Stoianova-Ivanova et al., 1974), are found in infected plant extracts.

**Figure 6:**
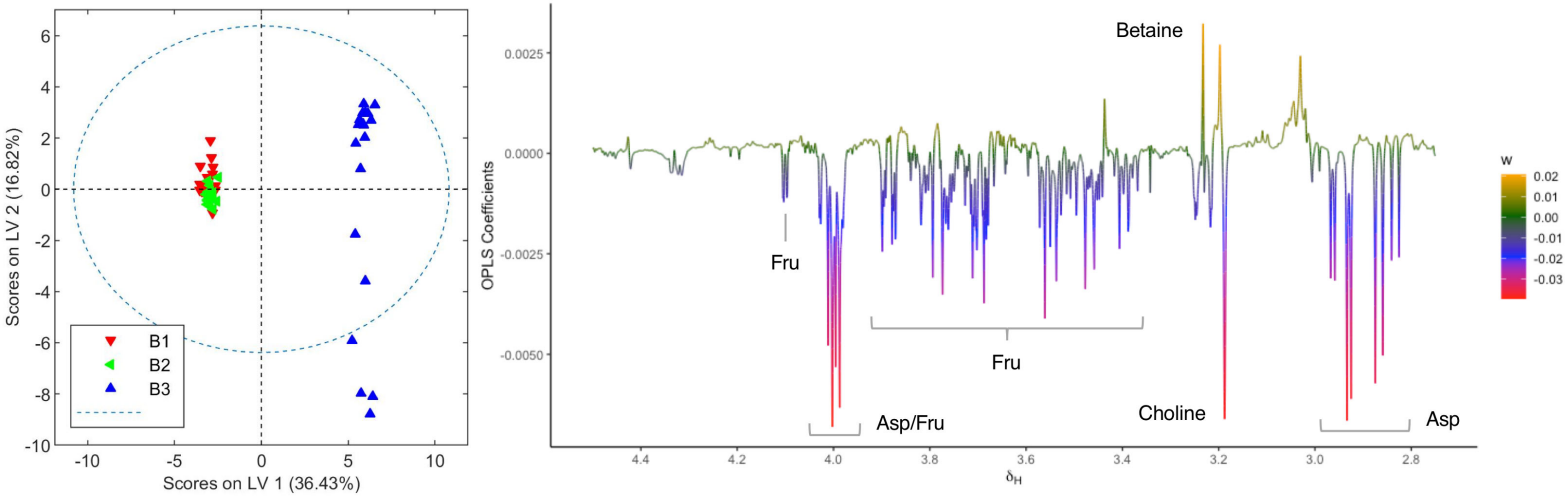
Score and loading factor plots obtained from the OPLS-DA between aqueous extracts of healthy and infected crops of chemovar B. The metabolites that differentiate the two groups are annotated in the loading factor plots. The R^2^Y and Q^2^Y coefficients for the model were 0.99 and 0.98, respectively, and the ROC curve had an AUC value of 1.00 (see **Figures S15**, **S16**).

The OPLS-DA model comparing aqueous extracts from the B1+B2 harvest versus those from B3 can also classify healthy and infected plants correctly (**Figure 6**). As observed in the loading factor plot, aspartate, choline, and fructose are in this case associated with healthy chemovar B specimens, while the levels of betaine are higher in infected plants.

## 4 Discussion

As stated in the introduction, our initial goal was to perform an exhaustive chemical characterization of a high-THCA and a high-CBDA *C. sativa* chemovars with medicinal potential through the determination of their metabolic profiles. Our results revealed a number of differences between the two varieties beyond their cannabinoid composition. This is an important finding since more than five hundred different compounds have been reported in the plant, some with potential therapeutic qualities, and combinations of the various secondary metabolites could determine both the final medicinal response as well as any adverse effects (Lowe et al., 2021; Procaccia et al., 2022). In fact, and despite the results from NMR-based metabolomic analysis of organic extracts presented in **Figures 2**, **3** parallel those obtained through HPLC (**Tables 1**, **2**), the study of aqueous fractions identified aspartate, betaine, choline, fructose, and quebrachitol as metabolites that can further discriminate between the two chemovars. The differences in sugar and aminoacid contents, which have been described by Choi and coworkers in their seminal report on the application of metabolomic profiling to the characterization of cannabis cultivars (2004a), could be employed to better fingerprint cannabis varieties and products intended for use as botanical drugs. This is also the case for quebrachitol, a long-known component of *C. sativa* (Adams et al., 1940; Groce and Jones, 1973). Indeed, variations in the concentration of this cyclitol have also been employed to address the phenotypical plasticity in other plants (Kortesniemi et al., 2017), making it a promising phytomarker for cultivar differentiation. Given the correlation between betaine levels and abiotic stress (Rhodes and Hanson, 1993), the identification of higher concentrations of this metabolite in high-CBDA chemovars suggest better adaptation to drought, high salinity, and low temperature conditions. In principle, this information would be instrumental to breeders in the development of plant varieties agronomically adapted to specific environmental conditions.

In addition to differences between varieties, analysis of the data from different harvests also allowed us to identify changes in the metabolome caused by fungal infection. As summarized in **Figures 5**, **6**, higher levels of CBDA, fructose, aspartate, and choline were observed in healthy plants from chemovar B, while increased levels of waxes and betaine were found in infected specimens. It is well known that biotic and abiotic stresses, and in particular bacterial and fungal infections, can lead to variations in the cannabinoid profile in *C. sativa* (Gorelick and Bernstein, 2017). While these variations were not evident when comparing the cannabinoid compositions of the three crops using data from standard HPLC methods, multivariate analysis of NMR data was highly sensitive and allowed to readily detect them. Indeed, CBDA was identified as one of the metabolites that can aid in the classification of healthy and diseased plants. While HPLC revealed a slightly lower concentration for this metabolite in the third crop relative to the first two (13.23% versus 14.30 and 14.38%), the variation is well-within quality specifications proposed for botanical drugs (Sarma et al., 2020). Furthermore, the full cannabinoidic profile obtained by HPLC was the same for the three crops. In agreement with their roles in stress response, the concentration of waxes and betaine was also higher in plants with signs of infection. The former compounds constitute one of the first lines of defense against microbes (Moyna and Heinzen, 2001; Arya et al., 2021). While larger alterations in wax composition could be expected in leaves, variations in its concentration in inflorescences are also likely upon fungal infection (Inada and Savory, 2011). Similarly, and taking into account its role as an osmoprotectant that counteracts the effects of reactive oxygen species and promotes membrane stabilization (Rhodes and Hanson, 1993), variations in betaine levels are consistent with the necrotic lesions observed on plant tissue affected by powdery mildew (Punja et al., 2019). As discussed above, variations in betaine concentration in plants are normally associated with abiotic stress. However, there is evidence that fungal infections can also lead to increases in the levels of this quaternary ammonium alcohol in other crops (Murray and Ayres, 1986; Aslanpour et al., 2016).

To summarize, our studies contribute to the holistic chemical characterization of *C. sativa* varieties with potential medicinal applications. Given the variability in the chemical composition of these plants and their extracts, this information is critical to assure the quality of botanical drugs and related products derived from them. In addition, the results presented above further showcase the suitability of metabolomic profiling as a tool for the rational classification of plant materials.

## Data availability statement

The raw data supporting the conclusions of this article will be made available by the authors, without undue reservation.

## Author contributions

SF: methodology, data curation, formal analysis, investigation, and writing–original draft. RC: data curation and formal analysis. AL-R: data curation, formal analysis, and investigation. PR: data curation, formal analysis, and investigation. IC: project administration, funding acquisition, and writing–review and editing. CG-C: conceptualization, methodology, project administration, funding acquisition, and writing–review and editing. GM: conceptualization, methodology, project administration, and writing-review and editing. All authors contributed to the article and approved the submitted version.
