# Supplementary material for: Beyond cannabinoids: Application of NMR-based metabolomics for the assessment of Cannabis sativa L. crop health
Source: Front Plant Sci. 2023 Mar 22;14:1025932. doi: 10.3389/fpls.2023.1025932 (PMC10075229; doi:10.3389/fpls.2023.1025932)
Supplement: Supplementary file 1 [file DataSheet_1.docx]

Supplementary Material

**
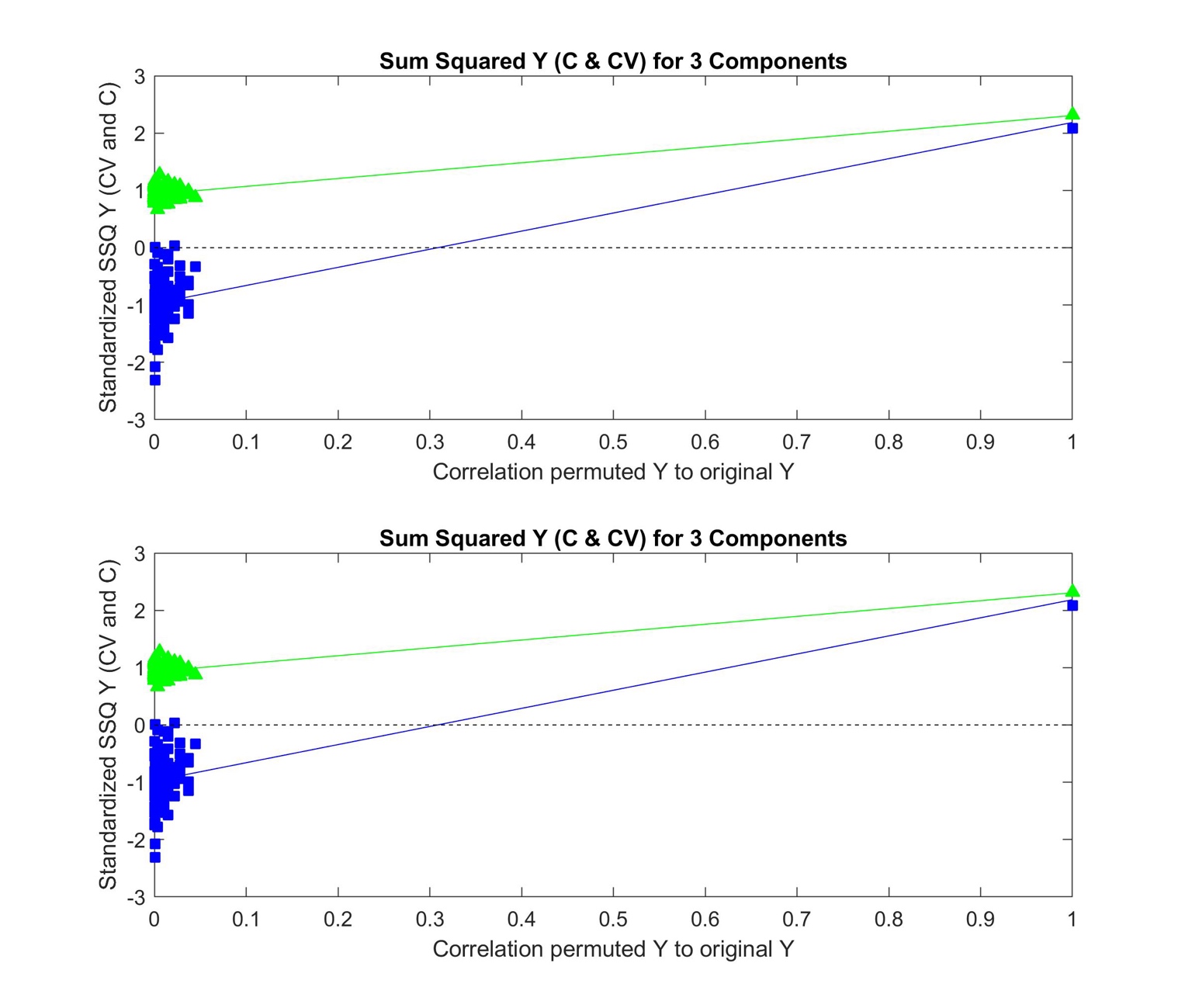
**

**Figure S1**. Permutation test plots for the OPLS-DA model comparing the organic extracts of chemovars A and B (R^2^Y = 0.99 and Q^2^Y = 0.99).


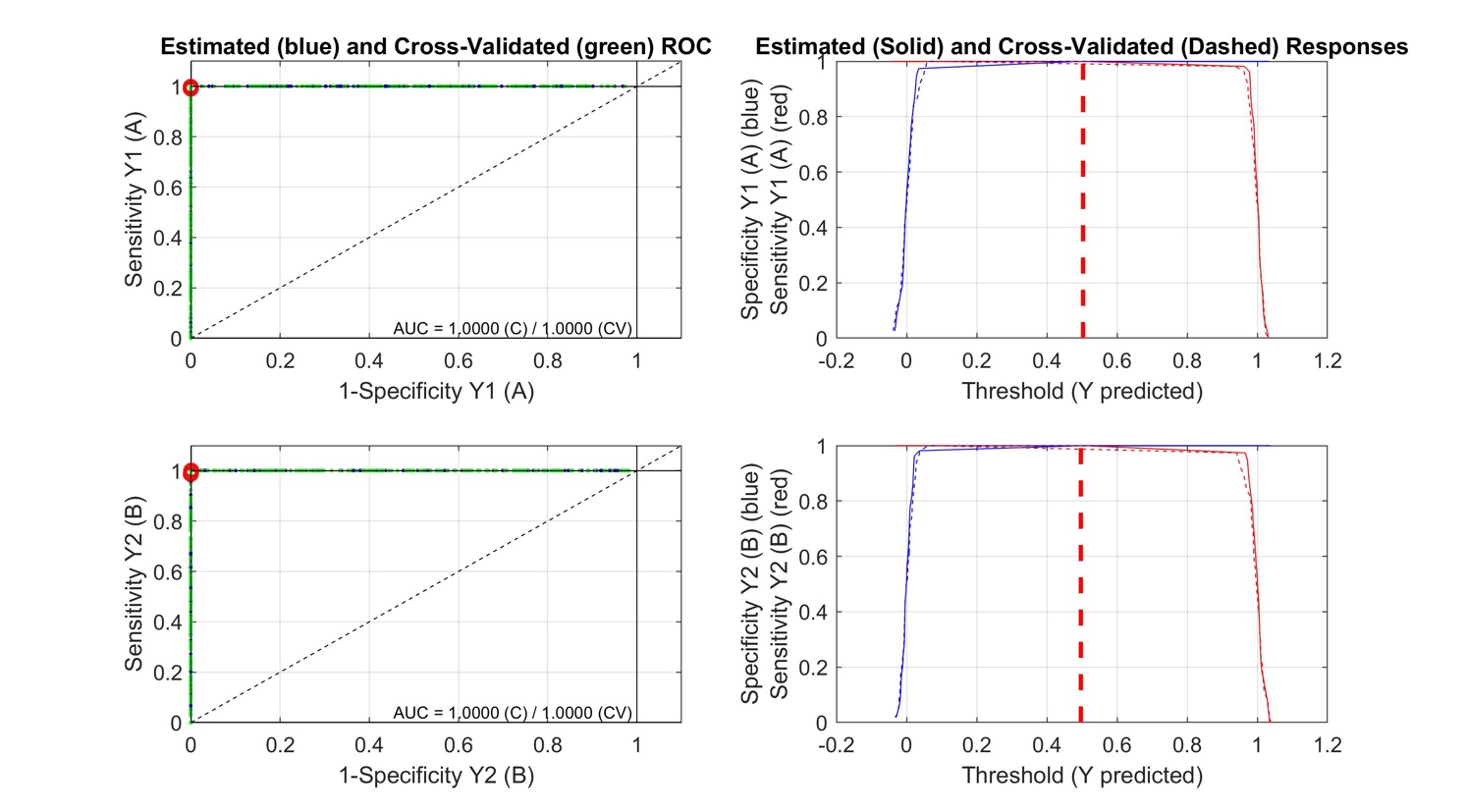


Figure S2. ROC analysis curves derived from the OPLS-DA model comparing the organic extracts of chemovars A and B (AUC = 1.00).

**Figure S3**. HSQC spectrum of a representative aqueous extract from chemovar B inflorescences. The ^1^H-^13^C correlation corresponding to the *N*-methyl groups in betaine is annotated.


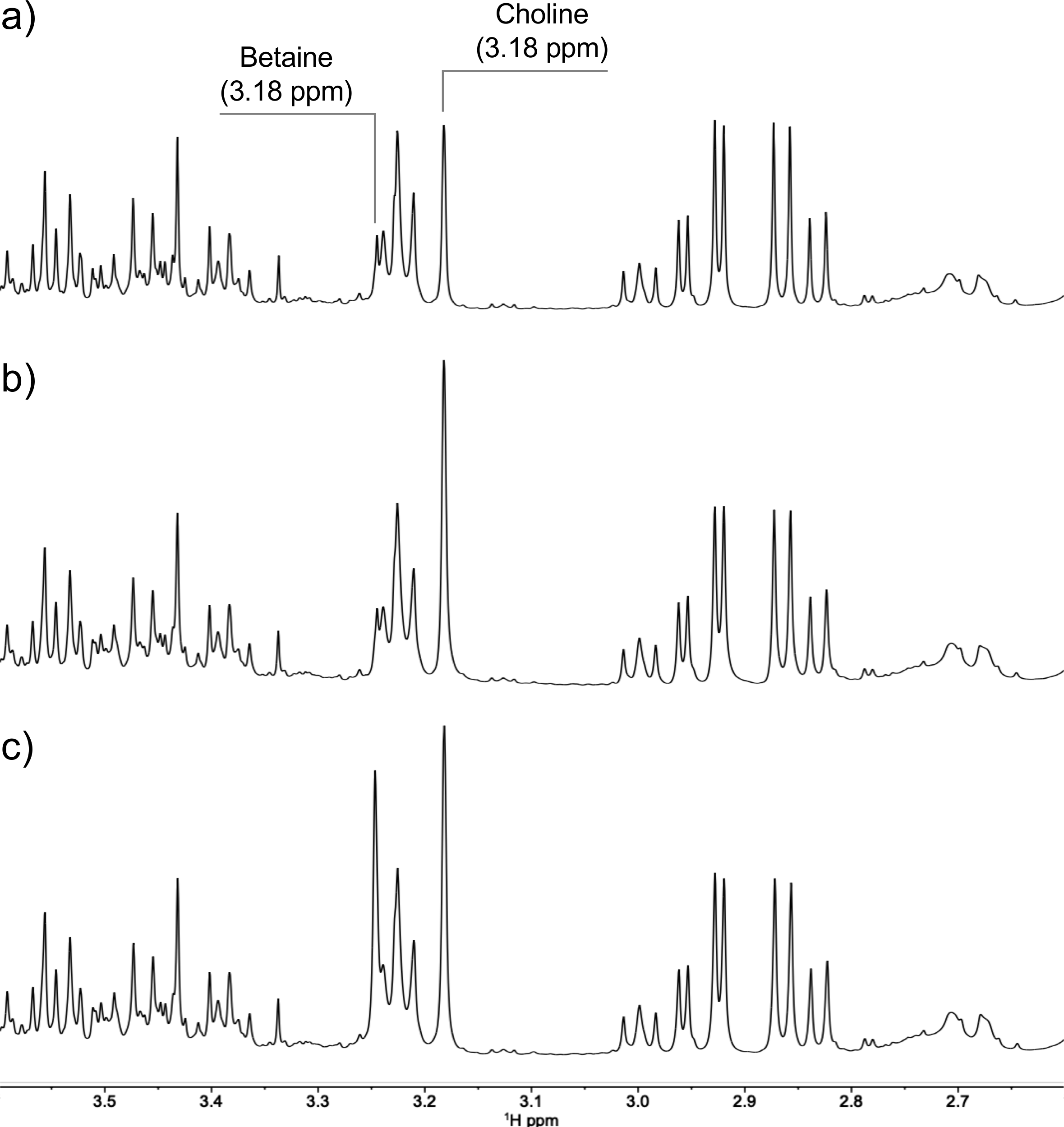


**Figure S4**. ^1^H NMR spectrum of a representative aqueous extract from chemovar B inflorescences indicating the resonances tentatively assigned to the *N*-methyl protons of choline and betaine at 3.18 and 3.23 ppm, respectively (a), and of the same sample after the successive addition of 10 μL aliquots of 50 mM choline (b) and betaine (c) standard solutions.

**
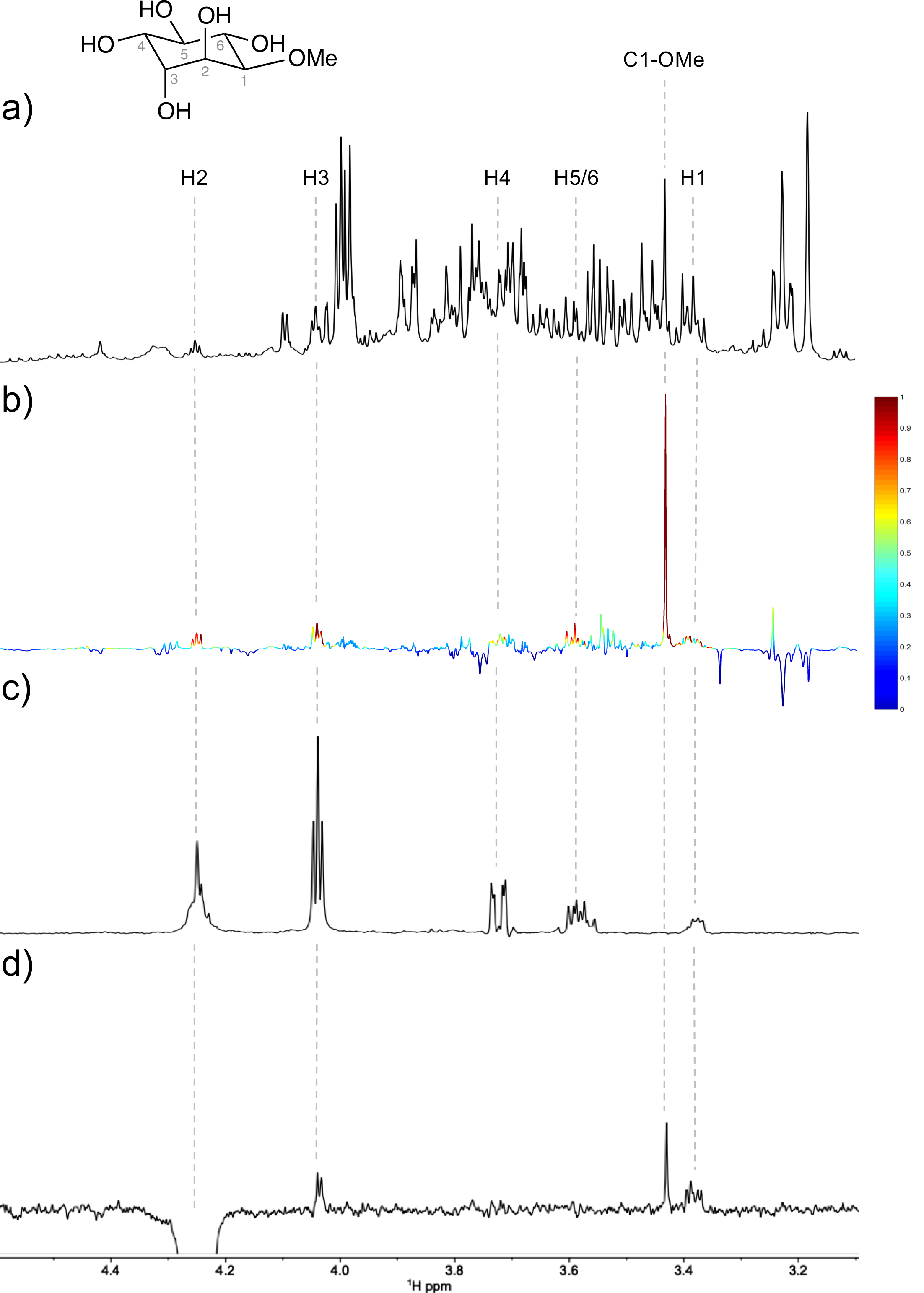
**

**Figure S5**. b) STOCSY spectrum obtained using the depurated aqueous extract ^1^H NMR data matrix employed in the multivariate analyses and the signal at 3.44 ppm as a driver peak. The STOSCY algorithm identifies peaks that have high covariance with a selected driver peak, and can help identify structural or biological correlations (Cloarec et al., 2005). The results are portrayed as a ^1^H trace that is colored using a continuous coloring scheme, and in which signals that hold high covariance with the driver peak appear red. Since all ^1^H signals in a given molecule vary their intensity proportionally throughout the data matrix, they all yield strong STOCSY correlations with the driver peak within the same molecule. c) 1D-TOCSY spectrum obtained on a representative aqueous extract from chemovar A inflorescences with selective excitation of the H2 resonance at 4.25 ppm. Only the signal at 3.44 ppm, which corresponds to the C1-OMe proton signal and is not part of the cyclitol ring spin system, is absent in the correlation spectrum. d) 1D‑NOESY spectrum obtained on the same sample with selective inversion of the H2 resonance at 4.25 ppm. Dipolar couplings to the C1‑OMe, H1, and H3 protons with enhancements of 1.07, 1.31, and 1.12%, respectively, are observed. a) Standard 1D ^1^H NMR spectrum of the same aqueous sample presented for comparison. ^1^H signals corresponding to quebrachitol and are annotated.


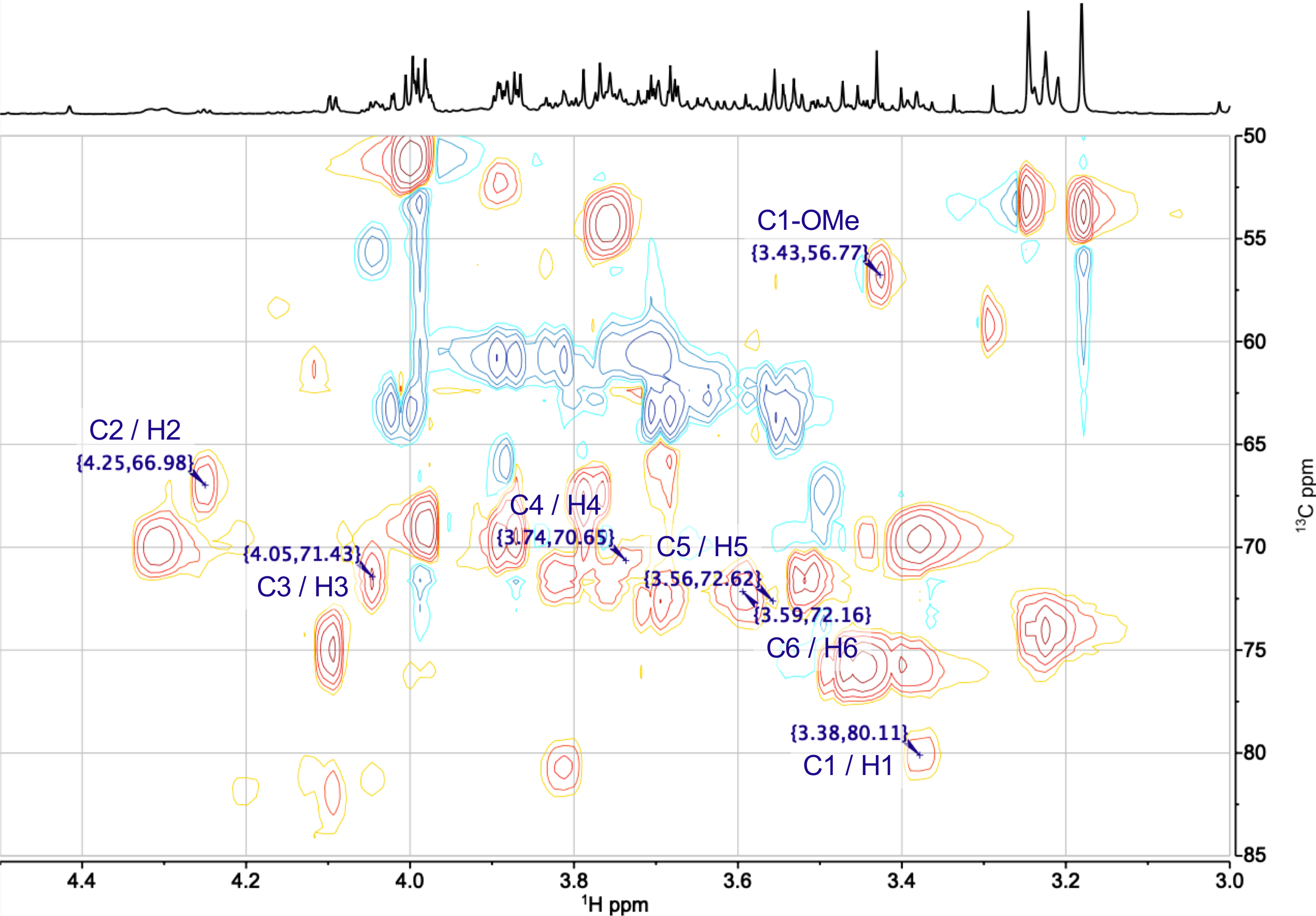


**Figure S6**. HSQC spectrum of a representative aqueous extract from chemovar A inflorescences. ^1^H‑^13^C one bond correlations corresponding to quebrachitol are annotated.

**
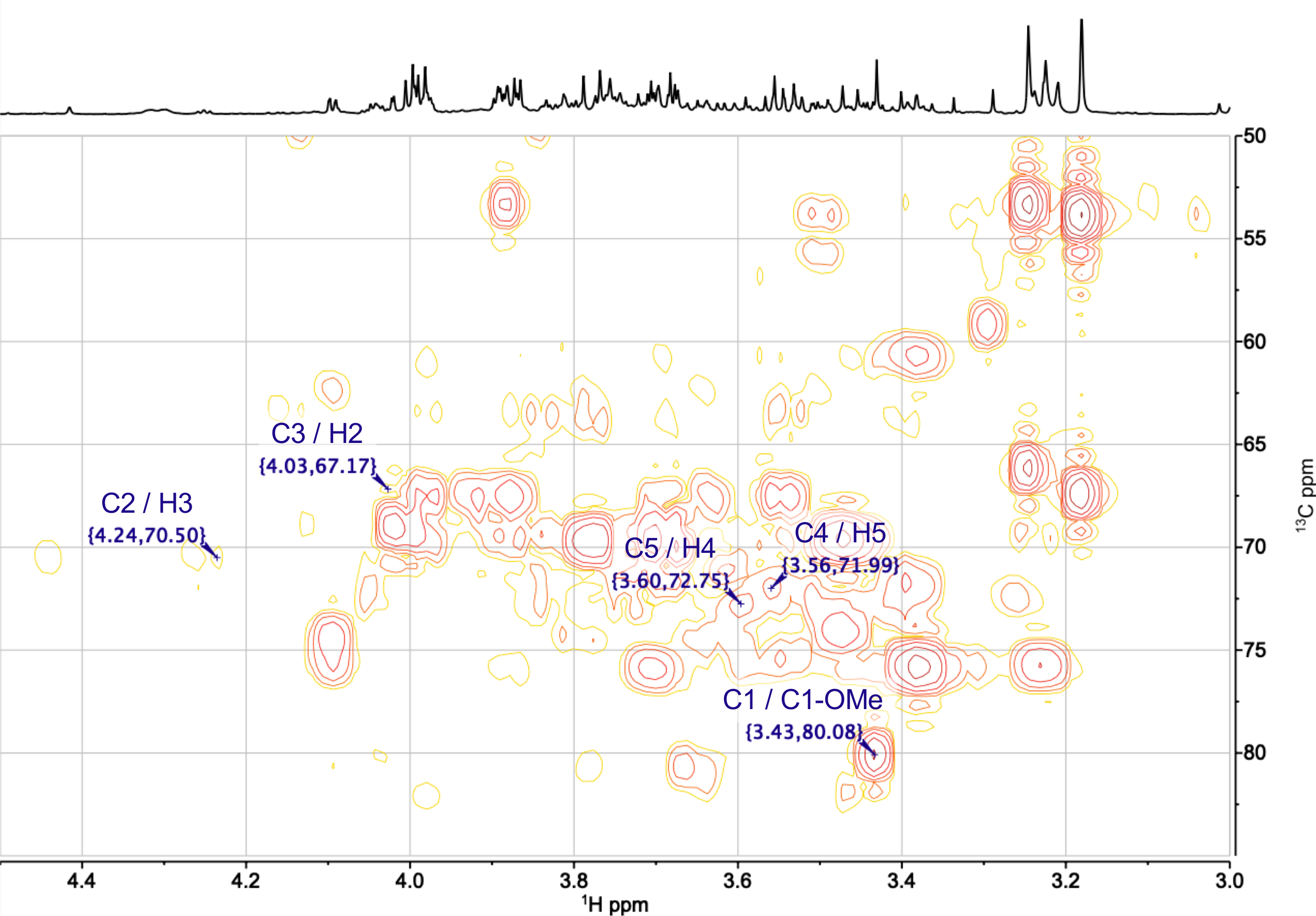
**

**Figure S7**. HMBC spectrum of a representative aqueous extract from chemovar A inflorescences. ^1^H-^13^C long range correlations corresponding to quebrachitol are annotated.

**Table S1**. ^1^H and ^13^C assignments for quebrachitol.

| **Position** | **^1^H** | **^13^C** |
| --- | --- | --- |
| 1 | 3.38 | 80.1 |
| 2 | 4.24 | 67.0 |
| 3 | 4.04 | 71.4 |
| 4 | 3.73 | 70.4 |
| 5 | 3.57 | 72.4 |
| 6 | 3.59 | 71.8 |
| C1-OMe | 3.44 | 56.8 |

**Figure S8**. HSQC spectrum of a representative aqueous extract from chemovar A inflorescences. The ^1^H-^13^C correlation corresponding to the methyl groups in choline is annotated.


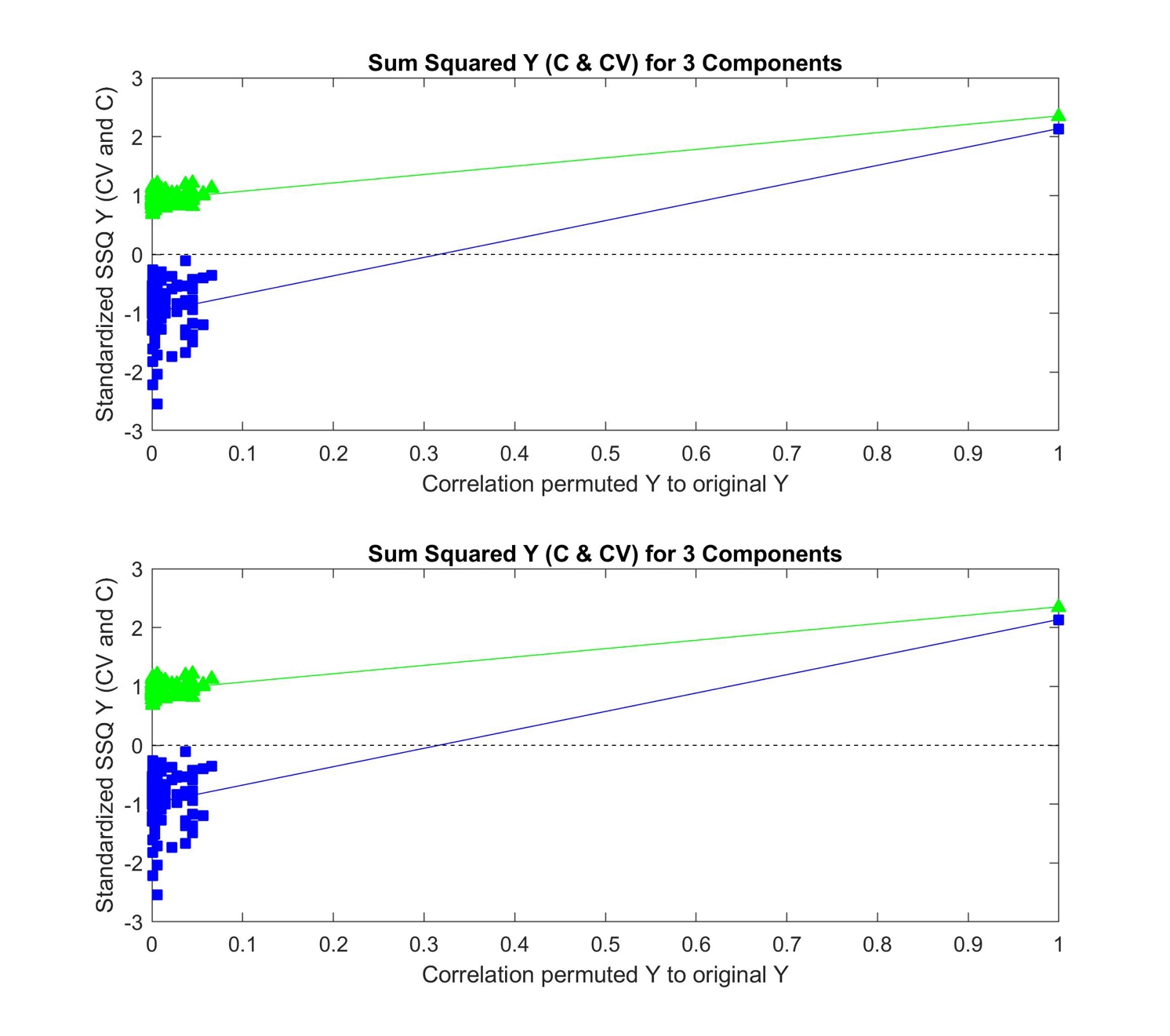


**Figure S9**. Permutation test plots for the OPLS-DA model comparing the aqueous extracts of chemovars A and B (R^2^Y = 0.91 and Q^2^Y = 0.83).


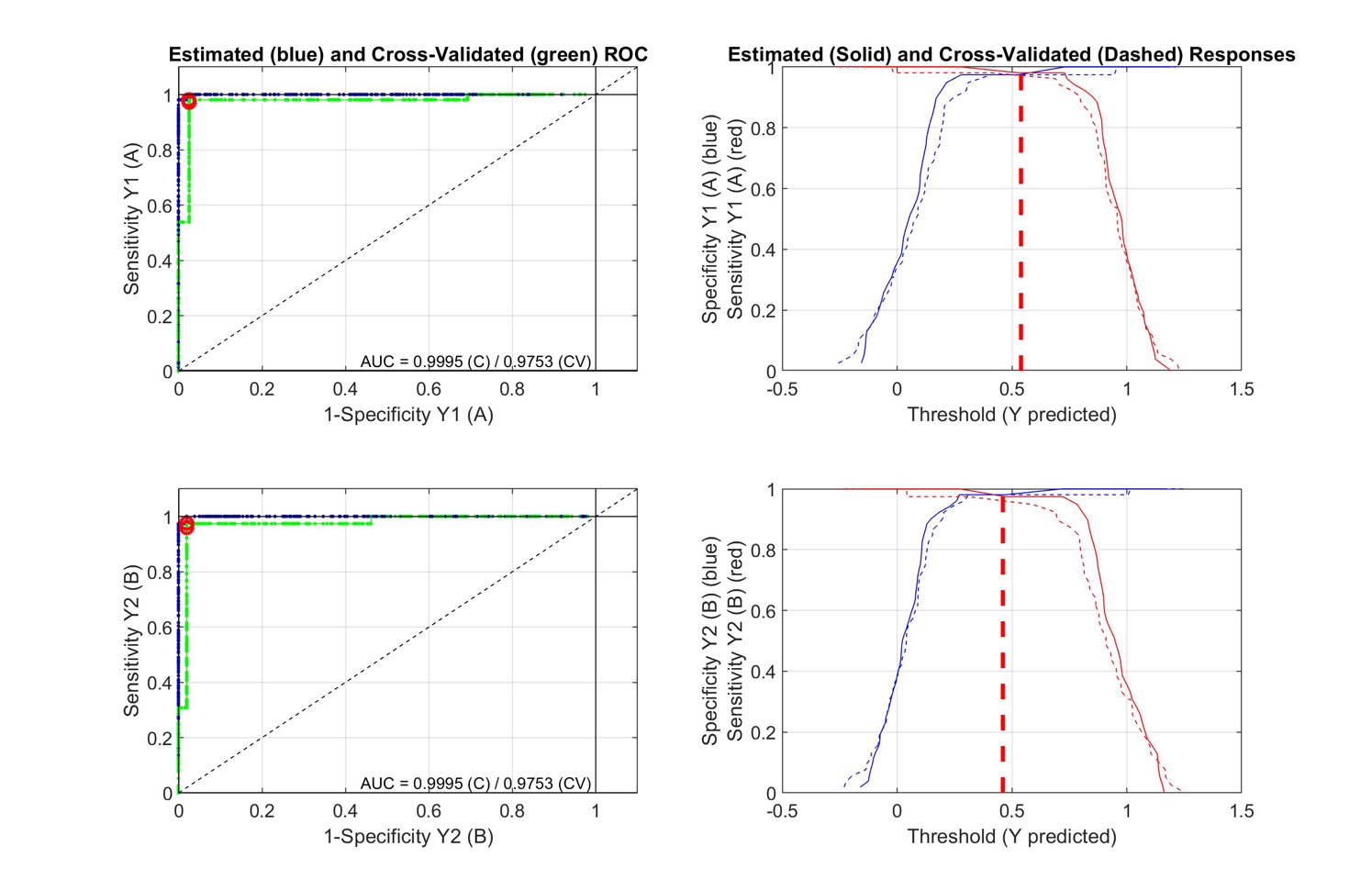


**Figure S10**. ROC analysis curves derived from the OPLS-DA model comparing the aqueous extracts of chemovars A and B (AUC = 0.98).

**
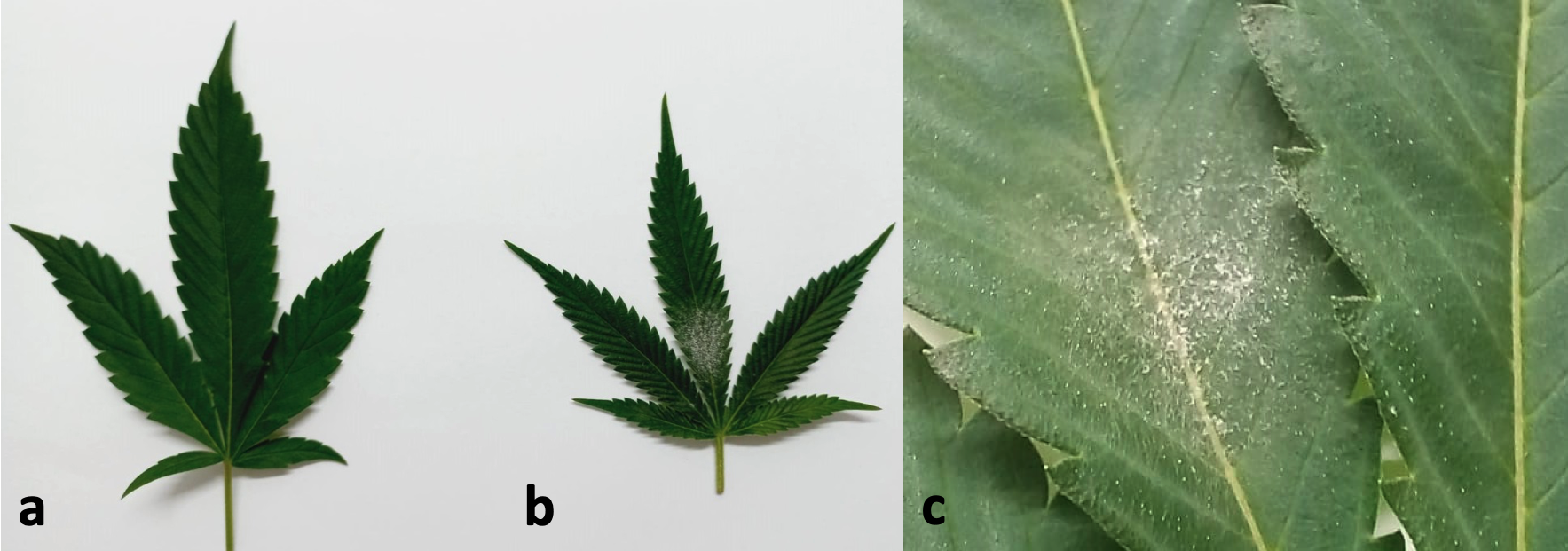
**

**­­Figure S11**. Healthy (a) and infected (b) *C. sativa* leaves. Magnification of the affected area clearly shows powdery mildew as white colonies on the upper surface of leaves (c). The mycelium of the pathogenic fungus is amphigenous, thin, and white, forms small to moderately large patches, is persistent on the upper surface of the leaf, and is less conspicuous and often evanescent on the lower surface. These characteristics are consistent with what was described for the genus *Golovinomyces* (Brochu et al., 2022; Punja et al., 2019; Szarka et al., 2019).

**
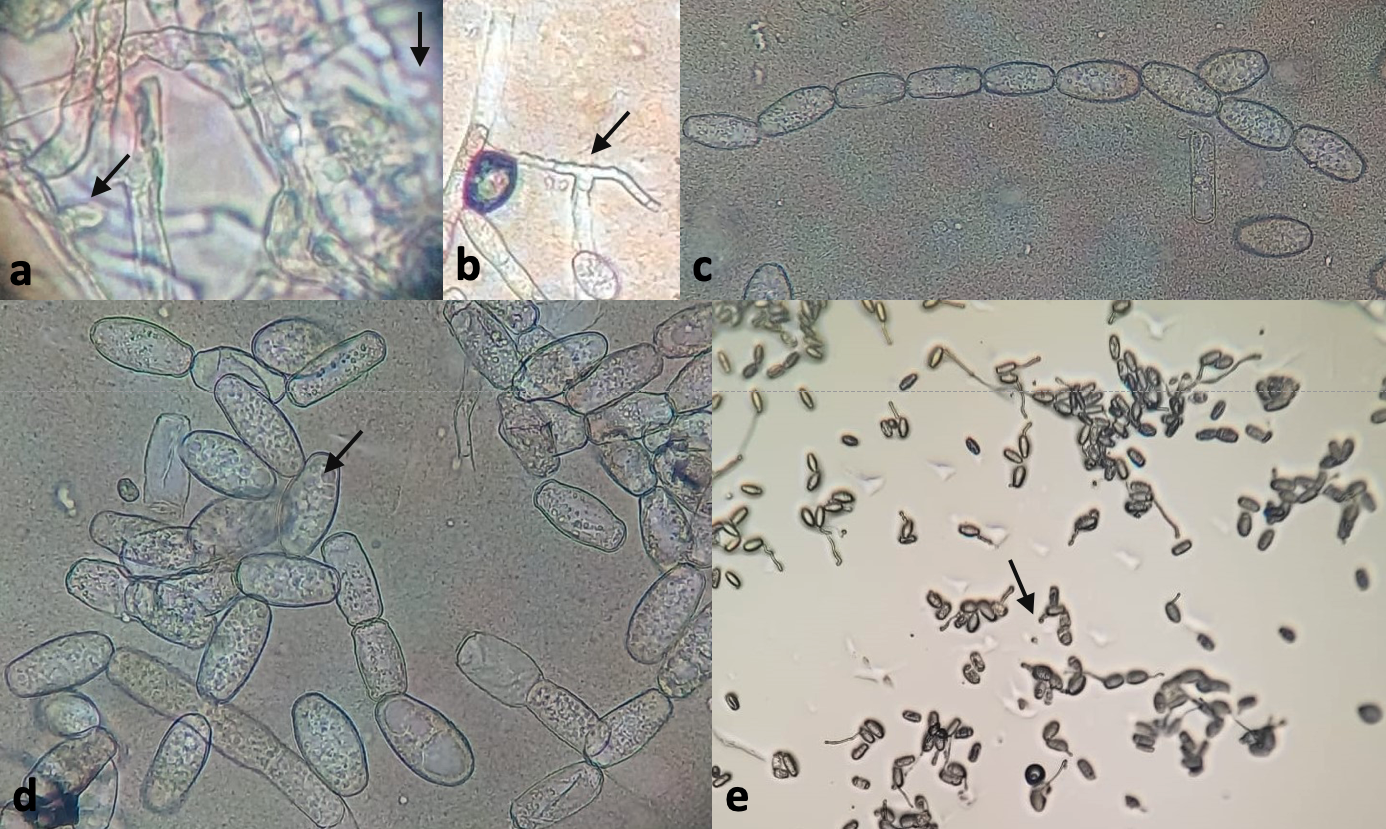
**

**Figure S12**. Microscopic images of the powdery mildew pathogen on leaf surfaces of cannabis plants, showing hyphae and hyphal papillate appressorium (marked with arrows, a), conidiophore with foot cell (b), conidia in chains (c), conidia with oil-like drops (marked with arrows, d), and conidial germination with germ tubes ending in swollen appressoria (marked with arrows, e). Hyphae were septate, thin-walled, smooth and hyaline, with the presence of hyphal appressoria papillate-shaped. Conidiophores were long with foot cells and the basal conidium forming spores in chains that is a characteristic of the genus *Golovinomyces* (Pépin et al., 2018). Oil-like drops were present within conidia although no distinct fribosin bodies were observed. Conidial germination was apical to sub-apical, with germ tubes ending in swollen appressoria. Chasmothecia were not seen. These microscopic characteristics agree with that described for the genus *Golovinomyces* (Qiu et al., 2020; Rajmohan et al., 2022; Weldon et al., 2020).


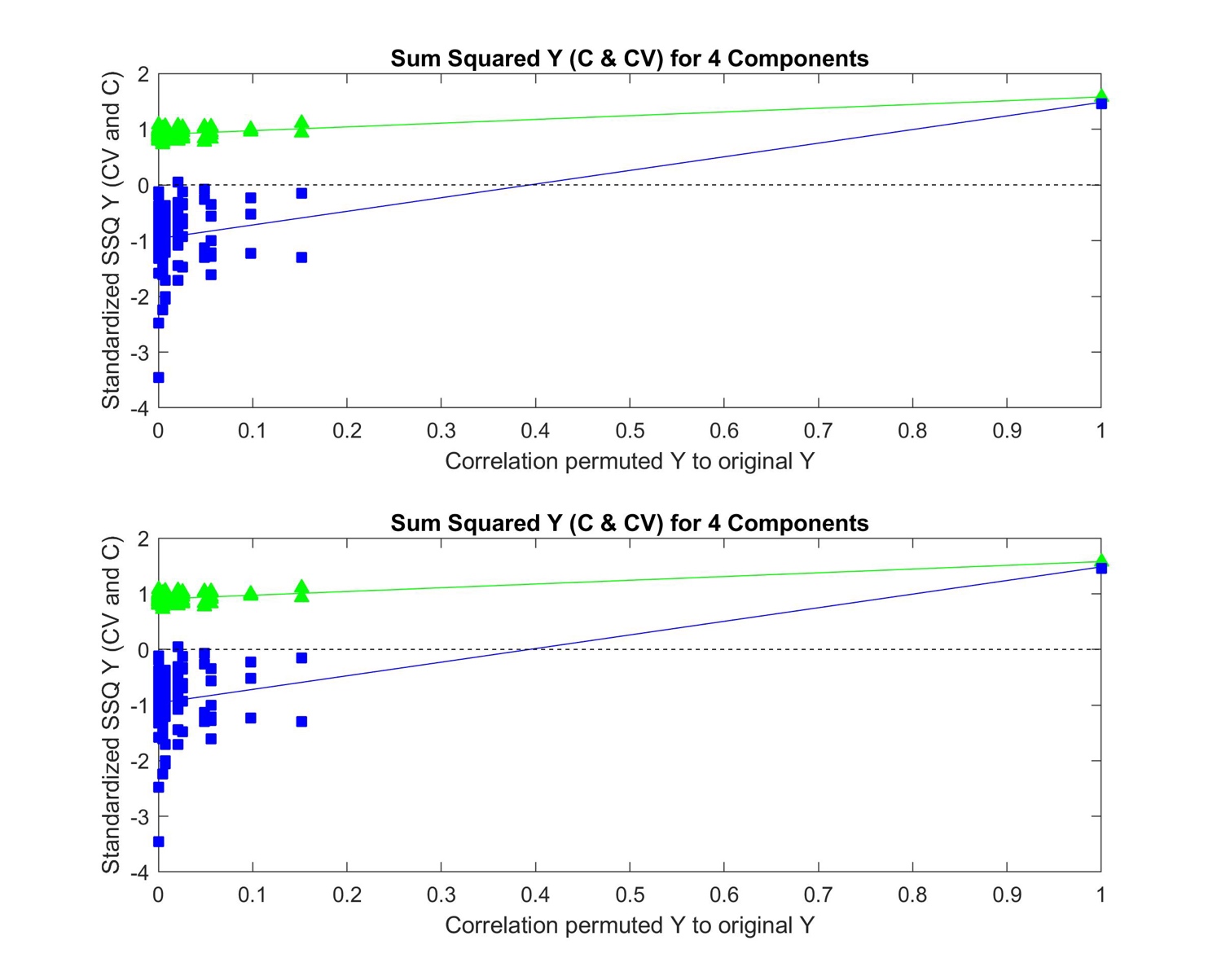


**Figure S13**. Permutation test plots for the OPLS-DA model comparing the organic extracts of healthy (B1+B2) and infected (B3) crops (R^2^Y = 0.98 and Q^2^Y = 0.91).


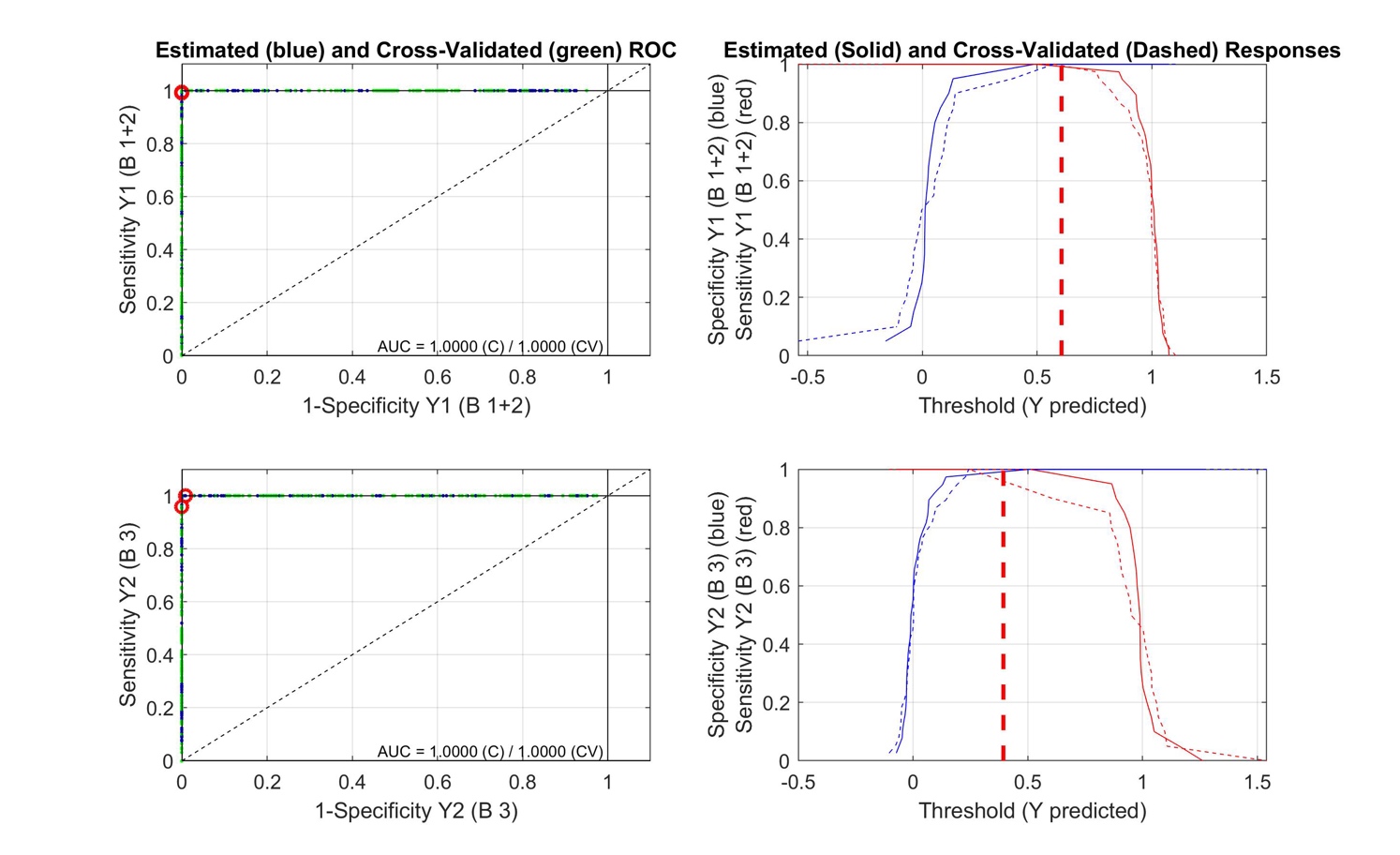


**Figure S14**. ROC analysis curves derived from the OPLS-DA model comparing the organic extracts of healthy (B1+B2) and infected (B3) crops (AUC = 1.00).


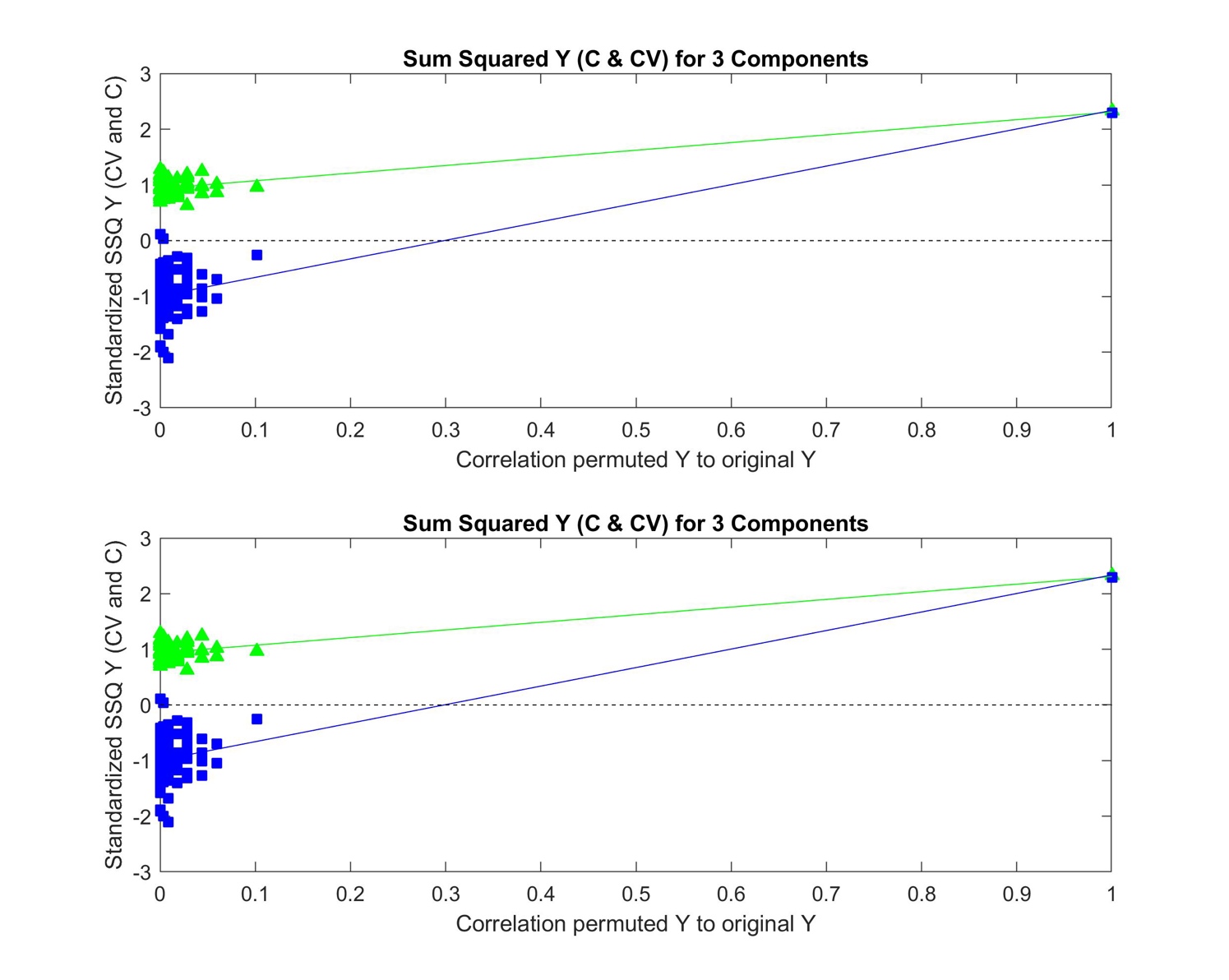


**Figure S15**. Permutation test plots for the OPLS-DA model comparing the aqueous extracts of healthy (B1+B2) and infected (B3) crops (R^2^Y = 0.99 and Q^2^Y = 0.98).


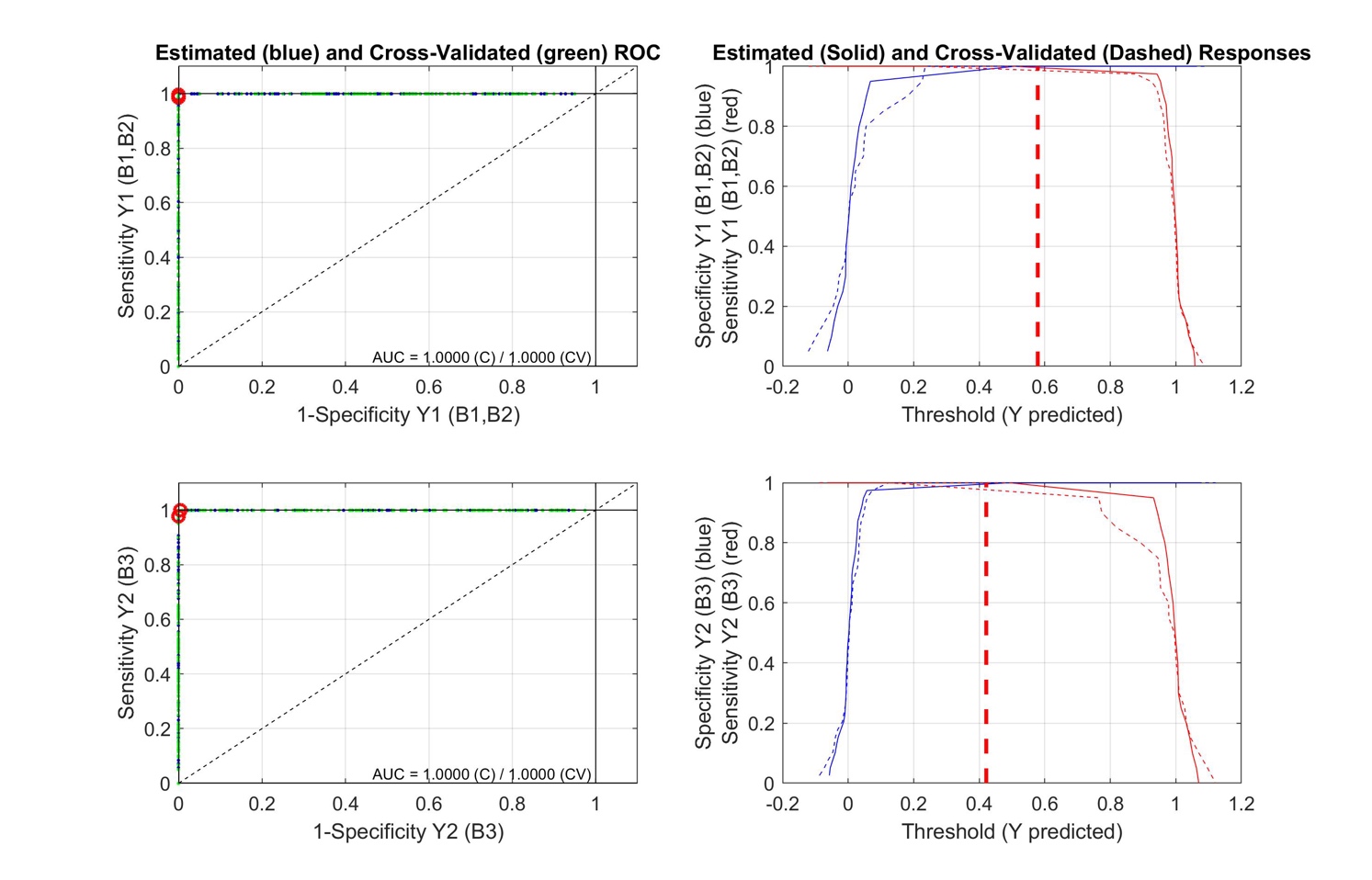


**Figure S16**. ROC analysis curves derived from the OPLS-DA model comparing the aqueous extracts of healthy (B1+B2) and infected (B3) crops (AUC = 1.00).

**References**

Brochu, A.-S., Labbé, C., Bélanger, R., Pérez-López, E., 2022. First Report of Powdery Mildew Caused by *Golovinomyces ambrosiae* on *Cannabis sativa* (Marijuana) in Quebec, Canada. Plant Dis. 106, 2747. https://doi.org/10.1094/PDIS-02-22-0350-PDN

Cloarec, O., Dumas, M.-E., Craig, A., Barton, R.H., Trygg, J., Hudson, J., Blancher, C., Gauguier, D., Lindon, J.C., Holmes, E., Nicholson, J., 2005. Statistical Total Correlation Spectroscopy:  An Exploratory Approach for Latent Biomarker Identification from Metabolic ^1^H NMR Data Sets. Anal. Chem. 77, 1282–1289. https://doi.org/10.1021/ac048630x

Pépin, N., Punja, Z.K., Joly, D.L., 2018. Occurrence of Powdery Mildew Caused by *Golovinomyces cichoracearum sensu lato* on *Cannabis sativa* in Canada. Plant Dis. 102, 2644–2644. https://doi.org/10.1094/PDIS-04-18-0586-PDN

Punja, Z.K., Collyer, D., Scott, C., Lung, S., Holmes, J., Sutton, D., 2019. Pathogens and Molds Affecting Production and Quality of Cannabis sativa L. Front. Plant Sci. 10.

Qiu, P.-L., Liu, S.-Y., Bradshaw, M., Rooney-Latham, S., Takamatsu, S., Bulgakov, T.S., Tang, S.-R., Feng, J., Jin, D.-N., Aroge, T., Li, Y., Wang, L.-L., Braun, U., 2020. Multi-locus phylogeny and taxonomy of an unresolved, heterogeneous species complex within the genus Golovinomyces (Ascomycota, Erysiphales), including G. ambrosiae, G. circumfusus and G. spadiceus. BMC Microbiol. 20, 51. https://doi.org/10.1186/s12866-020-01731-9

Rajmohan, N., Price, D.C., Buckley, R.J., Komar, S.J., Bamka, W.J., Petit, E.A., Cabrera, R.I., Gianfagna, T.J., Simon, J.E., Wyenandt, C.A., 2022. First Report of Powdery Mildew Caused by *Golovinomyces ambrosiae* on Industrial Hemp in New Jersey. Plant Dis. 106, 2534. https://doi.org/10.1094/PDIS-12-21-2657-PDN

Szarka, D., Tymon, L., Amsden, B., Dixon, E., Judy, J., Gauthier, N., 2019. First Report of Powdery Mildew Caused by *Golovinomyces spadiceus* on Industrial Hemp ( *Cannabis sativa* ) in Kentucky. Plant Dis. 103, 1773–1773. https://doi.org/10.1094/PDIS-01-19-0049-PDN

Weldon, W.A., Ullrich, M.R., Smart, L.B., Smart, C.D., Gadoury, D.M., 2020. Cross-Infectivity of Powdery Mildew Isolates Originating from Hemp ( *Cannabis sativa* ) and Japanese Hop ( *Humulus japonicus* ) in New York. Plant Health Prog. 21, 47–53. https://doi.org/10.1094/PHP-09-19-0067-RS
